# Book Reviews

**Published:** 1814-11

**Authors:** 


### CRITICAL ANALYSIS

### *OF RECENT PUBLICATIONS*

IN THE

### DIFFERENT BRANCHES OF PHYSIC, SURGERY, AND MEDICAL PHILOSOPHY.

***Thoughts on Puerperal Fever, and its Cure by Spirits of Turpentine, illustrated by Cases in the Lying-in Hospital, Dublin; also, Cases of Inflammation and Spasm, cured by the internal and external Exhibition of that Medicine; with Correspondence on the Subject.*** By JOHN BRENAN, M.D. 8vo. pp. 24. Underwood, 1814.

DAILY observation proves to us the little dependence that is to be placed on medical reasonings; the data upon which we proceed are so unstable in themselves, that it cannot be a matter of surprise to witness the daily fluctuation of opinions seemingly the most indestructible. Upon the degree of credibility due to our author's statements, we cannot give an opinion. That must rest solely

 on on his character for veracity, or talent for observation. But, admitting the facts here adduced, which we see no reason to doubt, the axe is at once laid to the root of our preconceived notions on the subject of puerperal fever, and of the remedies considered to be necessary for its cure. A disease confessedly inflammatory would naturally be attacked by bleeding and cathartics. Who would have imagined the oil of turpentine, generally supposed to be a highly stimulating remedy, would have proved a specific for it? Should this turn out to be really the case, for "adhuc sub judice lis est," we shall hail the discovery as one of great importance to science; not considering it in connexion with puerperal disease alone, but as opening to us a wide field for observation, and as exemplifying in a striking degree the slender foundation on which the doctrines of the schools are erected.

We regret that the pamphlet before us has been drawn up in a manner so loose as to leave us quite in the dark respecting the *modus operandi* of the substance recommended, or whether any sensible operation is necessary to the relief from pain. Should these observations meet the eye of Dr. B., we hope he will furnish us with further information. We could have wished also to have had a more explicit detail of the symptoms of the several cases related of the successful application of his remedy. Dr. B. considered the cases to be inflammatory. Are the medical gentlemen of Dublin agreed on this subject? Was the disease inflammatory, or *puerperal colic?*

The pamphlet refers to some feelings of irritation between the doctor and some other gentlemen, but we are sorry to find the cause of them has been omitted in this impression; not that we are curious to pry into transactions of a private nature, nor can we justify the publication of libellous opinions affecting the character of individuals; but it is important to know whether the personalities were of a private origin, or the consequence of a refusal on the part of our author's brethren to admit the conclusions deduced from the cases under his observation.

The Puerperal Fever appeared, in the month of December, 1812, at the Lying-in Hospital of Dublin, in great force, and the mortality it occasioned was general; whole wards were swept away; and the medicines given seemed rather to increase than mitigate the disease. The gentlemen connected with the hospital made use of all those means in general estimation for the cure of puerperal fever. The physicians of note in the city very humanely contributed their assistance, and still the mortality shewed no abatement from the opposition of all the learning and experience of those gentlemen; some of whom, as teachers of medicine and writers on disease, are justly esteemed the ornament of their profession, and of high authority through all Europe. From such sources it must have been expected that the sufferers would have done for them all that was ever done for persons so afflicted: and such was the case; for bleedings were tried, blisters were applied, purgations by different medicines were used; and, with all, the malady was no way arrested.

We

We select the author's cases as the only evidence on this subject.

"CASE I.—Margaret Rogers had been bled to the amount of 30 ounces, in two bleedings. She was then sitting up, not being able to bear a supine posture. She was vomiting green and yellow bile incessantly; and the sensibility of the abdomen was such, that she could not bear the most gentle touch of the finger thereon. One of the assistants told me, that he considered effusion to have taken place; and, as this woman was in a rapid state of dissolution, from which no woman similarly affected ever recovered, he wanted to know what I would give her. I told him spirits of turpentine. He started, and asked me, how much. I told him, a table-spoonful. This appeared to him madness: but, as the woman seemed dying, he said I might give a few drops, but that he would not stand by whilst I gave it. Accordingly, at the hour of two, p. m. I gave her three tea-spoonfuls of the spirits of turpentine, in a little water. At five o'clock I returned, and the countenance of the assistant expressed the event: he remarked that the woman was better; and he had the courage to stand by when I gave her a table-spoonful, which he was much astonished did not burn her. He asked her, what she thought it was. She replied, Geneva and water. At nine o'clock we visited her. She lay at her ease. She never vomited from the time she took the turpentine, her abdomen was flaccid, and quite insensible of pain on the pressure of it, though very violent. The next day some pains returned; she took the turpentine; and they ceased. She called for food, and complained of hunger. She never after felt any uneasiness in the region of the uterus. In about four days after she began to spit pus, her pulse sunk, and she died. She was of an asthmatic disposition, and had come from the Lock Hospital, where she took much mercury. The turpentine cured all the symptoms that threatened speedy death. She had no symptom of her puerperal complaint after, and I had put her death down to the bleeding.—This case has been objected to me as unfavourable. I cured all I proposed curing; all her puerperal symptoms disappeared: and I fancy the annals of midwifery does not furnish an instance of such relief, in such circumstances, or of any one cured, or even relieved, when the symptoms went to the extent of her case.

"CASE II.—The second case I interfered with, was Margaret Conolly, servant to Mr. Grogan, of Merrion-square. She had labour for two days, and got the fever: the usual remedy was tried, she was bled twice to the amount of thirty ounces: I suggested to the assistant, the application of the turpentine to her abdomen, which was tense and sore to an exquisite degree: he allowed me to apply it; for the fears he held about it, even as to its external use, were such, as that he would not venture to apply it himself, and he therefore allowed me the privilege of doing any good that might turn out from its application. I poured it on her abdomen, covered with flannel. In about three hours after I saw her, she said I had cured her: her abdomen was as flaccid as when in health, it could bear pressure pressure to any degree, and her breathing was easy. From this circumstance I saw plainly, that the difficult breathing, for which bleeding is so frequently used, arose from the pressure of the diaphragm upon the lungs, by the inflation of the abdomen, which never relieves the belly, and which always sinks the patient. I gave her two tea-spoonfuls of the turpentine in hot water and sugar, and she said it disagreed with her stomach, (and I think cold water is a better vehicle); the next day her symptoms returned, and she was bled in the morning eighteen ounces, and in the evening eighteen ounces: the day after she begged me to apply the remedy I did before to her; I did so without permission, but for humanity sake; her abdomen became flaccid again, and she felt great relief, and could bear any pressure on it; she said, the only thing she complained of was her heart, but that could not rise: she sunk, but with no distress in the region of the uterus; and after death her abdomen was flat. I put down her death to the bleeding. I must note, that I had no further interference in this case than one permissive act, which relieved her; and one assumed authority, which also relieved her. I think, and I believe they about her think, that, if I had treated her alone, I should have been as fortunate as I have been in what I call worse cases. She was in No. 6, in the inside ward.

"CASE III.—A woman in ward No. 7, her name I know not, she lay next bed to Mrs. Keefe's daughter, who died of the fever; she was seized with head-ach, sore abdomen, and a turning in her stomach: she complained severely, and moaned much. I gave her a table-spoonful of the oleum terebinthinæ, and a sup of water after it. In about fifteen minutes I came to her; she told me she got ease. On coming into the ward the next evening, I went to her bed, and missed her; she was sitting at the fire, very well.

"CASE IV.—A woman in No. 8, who lay next bed to Mr. Allen's servant, of Dame-street, who died, got the symptoms that were the usual forerunners of this disease. I contrived by stealth to give an ounce of the turpentine in some saline mixture. This abated all her pains, and the vomiting: and the consulting doctor, on coming the next day, said nothing was the matter with her. She went home that day; the symptoms returned; a person from the Hospital went to her, bled and blistered, and gave the usual remedies. She died vomiting green bile, with her belly swelled. In this woman I stopped all the symptoms. She lived in Gloster-place, and was a smith's wife.

"CASE V.—Mary Murray, wife to a soldier in the Fermnagh militia, from Kilnese, near Naas, was delivered on Saturday 12th of the month: on Tuesday the fever set in with her most violently. I was allowed a latitude with this woman, on account of the surprising efficacy of the medicine with Rogers. She had a severe cough, and every time she coughed she screeched with the anguish of her belly, which was insufferably painful to the touch. I applied the turpentine to her abdomen, and gave her a table-sponful of it in water and sugar. The next day she was free from pai, and able to to eat bread and milk for breakfast: she took a drink of cold milk, and got ill as ever. I repeated the turpentine, and applied it to her abdomen. The next day she ate stirabout, and got a relapse from cold beer. She continued very ill till Monday, when the doctor showed her to me as a forlorn case. He showed me the blackness of her hands, which he said was the sure forerunner of death, and that Denman said no woman ever recovered that had it. Another doctor saw her, and agreed that she was one of the patients then moribund. Nothing was ordered for her this day, I suppose from the dreadful state she was in. This was Monday; she was sitting up, vomiting green bile. I gave her an ounce of the turpentine, and repeated it in an hour, and applied it to her belly. The next morning I found her asleep. On Tuesday I gave her castor oil, tinctura sennæ, and two drachms of the turpentine in the draught. This purged her much. On Wednesday she breakfasted on stirabout and milk; on Thursday did the same; on Friday she sat all day at the fire, and was put with the next case I shall mention, to sleep in one bed. On Saturday she requested to be left in the Hospital, and on Sunday she walked home to Berwick-street with her child in her arms. This woman was never bled.

"CASE VI.—Bridget Cullen, servant to Mr. Ennis, of Kingston, near Rathfarnham, was delivered of twins, on Saturday, 12th of February: she had hard labour, and took ill on the couch. On Wednesday following she got severely ill with head-ach, turning of her stomach, and continued so all night; the next day she became a matter of serious consideration, and she had the turpentine applied to her belly by means of flannel sopped in it, and got a table-spoonful by the mouth. In about two hours after she began to roar with the pain of her abdomen: the flannel was removed, and it acted as a severe rubefacient: in some hours after she felt herself at ease: the next day her pains returned, and she got the turpentine internally, a table-spoonful; she occasionally got it for three or four days; and on the 25th she was put into the same bed with Mary Murray, above mentioned, and on the 26th went home very well. This is a very strongly marked case; and it must be here noted, that she was not bled."

"The above cases I treated in the course of about three weeks, in the Lying-in Hospital. I hope the efficacy of the medicine is as fully established to be a specific by these, as by any given number: but I shall mention a few other instances wherein turpentine was exhibited with equally wonderful effect.—Mary-Anne F. had heated herself by washing, and to cool herself she stood with her bed-gown open before a window, and a cold breeze; in a few hours after she was seized with violent pain in the abdomen, and showed all the symptoms of violent inflammation. Her face was livid, pulse small, and panting for breath. I chanced to come into the house, and found her as ill as I thought possible. I bled her to the amount of twelve ounces, and went to the next apothecary and got some spirits of turpentine: I gave her a table-spoonful undiluted, and had her stuped.

stuped. I repeated the turpentine in about two hours, when the violence subsided, and gradually vanished. She was perfectly well in two or three days. This case occurred in the house of Dr. Wade, the celebrated botanist; a gentleman to whose liberality I am singularly indebted for furthering the reputation of my discovery.

"A child of the cook of the Chief Baron, living in Gordon's-lane, had been playing in the rain during Sunday. On Monday he was seized with pain in his bowels that convulsed him, and his mother thought he was dying. A lady, a friend of mine, in the house, sent for two ounces of the spirits of turpentine, and gave him half of it undiluted. On my coming in, I saw the child in great agony, his knees drawn to his mouth, and screaming with pain. They told me what was done, and said, that a few minutes ago his abdomen seemed drawn into his back. I immediately gave him the remainder of the turpentine, and put him into a warm bath, in which he was not five minutes when he called to be taken out of it; his bowels gave way; his pains ceased; he fell asleep. His fever manifested itself the next day. He lay for a week, and recovered by the ordinary treatment.

"The Rev. Mr. K., curate of Francis-street chapel, had been called to see a woman who had left the Lying-in Hospital through fear of the fever. She became so ill, that she sent for a clergyman. Mr. K. attended her, and declared that her anguish was such, from pain of the abdomen, that the persons present, nor himself, did not think she could live one hour. He knew I had left town that day; and, as she could not be worse, he took courage and gave her three tea-spoonfuls of the spirits of turpentine. He called next morning and asked, "Was the woman dead?" He was answered by herself, "No, sir; I feel myself quite easy. I got relief immediately from the medicine you gave me, and I slept well all the night."

"The infant of Mrs. B. had been visited by the attending accoucheur with another physician, both men of eminence. They declared the child to have trismus nascentium, or nine-day fits, confirmed. The jaw was quite stiff. I ordered the infant an injection, and added half a drachm of the spirits of turpentine. On seeing the child the next day, they were much astonished to see a change that they never saw in one with that disease: the trismus had disappeared, and the child got well."

The author adds three letters in confirmation of his opinion, which we subjoin.

*From Mr. Wigglesworth.*

"To John Brenan.

"As thou desirest that I should mention to thee my case, as nearly as I can recollect I shall do so.

"In the beginning of the first month I was in a delicate state of health; and, going from a warm room into a cold vault at night, I was seized the next morn, whilst in bed, with violent pain in the bowels. I took castor oil and different things for it; still the pain continued, and I was obliged to get out of bed and roll on the floor

 through through agony. At four o'clock thou camest to me, at which time thou found me in great anguish. Thou observed to Richard Pim, who was in the room, I will now show thee what turpentine can do.

"Thou ordered me two small bottles. At half past four I took one of them; I felt instant relief; I fell asleep, and never felt pain after. At eight o'clock thou came out from dinner: I told thee what I just mentioned. Thou observed, that the pain might return about four o'clock, and in that case thou directed me to take the other draught. I felt a slight return of the pain: I took the draught, as thou desired, and I never felt uneasiness after.

"*City Quay*, 35. JOHN WIGGLESWORTH."

*From Dr. Burns, of Glasgow.*

"*Glasgow, July* 20, 1814.

"SIR,—I have been favoured with yours of the 15th, together with your pamphlet on the use of turpentine in Puerperal Fever. In reply to your question, I have no hesitation in saying, that I believe the employment of that remedy in such cases is new.

"From observing the excellent effect of turpentine when given to my horse as a remedy for colic, and from a knowledge of its safety in the human species, when exhibited for the cure of tenia, I have considered it as a remedy deserving trial in spasmodic affections of the bowels: but I confess I would not have thought of employing it where there was any reason to suspect the existence of inflammation.

"Your trials prove, at least, that it is not injurious in that state of the intestine which exists in Puerperal Fever. But I should like to know whether it has ever been given early, and trusted to alone, with decided success.

"As I am anxious for the advancement of my profession, and deem it my duty to attend to every suggestion for improvement, I should wish to be favoured with any well-marked case, substantiating the efficacy of turpentine.

"I have the honour to be your very obedient servant,

"*To Dr. Brenan,* JOHN BURNS."

161, *Great Britain-street, Dublin.*"

*From Dr. Douglas.*

"50, *Great Britain-street; August* 2, 1814.

"SIR,—I beg leave to state, that I find more difficulty in commencing an answer to your letter on account of the terms in which you have spoken of myself, than from any hesitation I feel in lending my slender sanction to pronounce your discovery of the utility of turpentine, in the cure of Puerperal Fever, to be worthy of the attention of every physician who is not so absurd as to think that the profession has already arrived at an acmé of perfection.

"It is probable, however, that, like many other great discoveries, it will be little esteemed in its native country, until after it shall have received the sanction of foreign nations.

"There is scarcely a person who does not know that Peruvian bark is justly esteemed a specific for intermittent fever, but every person is not competent to administer it, as its doses and the periods of giving it must be varied according to circumstances. Neither can it be expected, although turpentine were admitted to be a specific for Puerperal Fever, that every accoucheur should know by rule of thumb, either the most proper periods, or the necessary repetitions for its administration. And, likewise, as there are required adjumenta to bark, in the cure of intermittent fever, so are there required adjumenta to turpentine in the cure of puerperal fever.

"If you expect to receive from me a detail of cases, you will be disappointed, as I do not keep notes of my practice. I have several cases, however, in my recollection, in which I administered the turpentine with the most gratifying results. And I can even positively assert, that I never yet ordered it to any patient who did not recover. If any person should suppose that my senses may have deceived me, I can refer him to living tests, some of whom can speak to what medicine they themselves, as well as I, attribute their recovery. I have the honour to remain your obedient servant,

"*To Dr. Brenan.* JOHN C. DOUGLAS."

---

*Two Cases, with Observations, demonstrative of the Powers of Nature to reunite Parts which have been, by accident, totally separated from the Animal System.* By WILLIAM BALFOUR, M.D. Edinburgh.

IN a preceding Review we had occasion to remark how little dependance is to be placed on medical reasonings. The pamphlet before us is an additional proof of the fact, and illustrates the necessity of guarding against the influence of theoretical opinions, unless clearly deduced from actual and indubitable observation. A short time ago, the man who in his practice calculated upon the *possibility* of uniting parts of an animal body *totally separated from each other*, would have been considered an ideot, and, should he attempt to prove the stability of his principles by argument, would pass unnoticed as a madman. There is not wanting both direct and collateral evidence in support of Dr. Balfour's statements; but we are so apt to discredit what we cannot explain, that the truth of all similar statements has been roundly denied.

Upon this principle Garangeot's story of the soldier whose nose was *bitten off and replaced with perfect union*, is ridiculed by Mr. John Bell; but let it be observed, this gentleman is equally sceptical with regard to the generally-admitted fact of the union of the tooth with the cock's comb, satisfactorily demonstrated by Mr. John Hunter, and the proof of which is to be found in many anatomical museums.*

* Since writing the above we understand Mr. Bell, in a subsequent edition of his book on Wounds, has retracted the latter of these opinions.

Mr.

Garangeot's case is thus related by him, and is intended to show the possibility of uniting wounds of cartilage.—"Sept. 26, 1724, a soldier, fighting with one of his companions, had the *whole cartilaginous part of his nose bitten off.* His adversary perceiving that he had a bit of flesh in his mouth, spat it out, and in a fury trampled upon it. The soldier picked up the end of his nose, threw it into the shop of a Mr. Galin, and ran after his enemy. In the mean time, Mr. Galin examined the amputated part, and, as it was covered with mud, he washed it at the fountain. The soldier returned to have it dressed. Mr. Galin replaced and retained it in its position by plasters. On the fourth day I dressed it myself, *and saw that the parts were completely united.**"

Candour obliges us to confess that we also, like the herd of our unthinking neighbours, "slow of heart to believe," were disposed to join Mr. Bell in giving the lie to this narrative, until similar instances forced themselves on our notice, accompanied with such marks of truth that the most stubborn scepticism could not fail to admit. At one of the meetings of the Westminster Medical Society held last year, two cases were related of this kind. To one of them in particular we may refer, not only because the facts were notorious to many individuals of the profession who were eye-witnesses of it, but because we are enabled, through the kindness of Mr. Kirkup, the present house-surgeon of St. Bartholomew's Hospital, to record it in our pages with sufficient evidence of its truth.

"A butcher," says Mr. Kirkup, "whose name I never knew, and whose address I am so far ignorant of, that I can only say he *then* lived somewhere about Islington, told me he had chopped a portion of his fore finger off (the division was about the middle of the second phalanx). On inquiring more particularly into the circumstances of the case, he stated that it was done about a month before, at eleven o'clock in the morning; that he wrapped the detached portion in a piece of paper, and brought it to the hospital at four in the afternoon, when Mr. Titler, a dresser of Sir James Earle's, applied together the surfaces occasioned by the division, and kept them in apposition by slips of adhesive plaster, the effect of which mode of treatment was, that union took place, and he eventually recovered the use of the whole finger. On enquiring of Mr. Titler, his account exactly agreed with that which the butcher had given. How long it is since this occurrence took place, I do not remember, but, as near as I can recollect, it must have been about August or September 1812, but I am far from being sure of the time."

Let it be observed, this cure was not effected in private. Like those related by our author, it is attended by marks which would inevitably lead to a disproval should it not be true, a circumstance which our respect for the honour of the profession, and the credit of the narrator, will not allow us for one moment to doubt.

---

* Traité des Operations de Chirurgie, tom. 3. Observation d'un nez *arraché,* jettée ensuite dans un ruisseau, & *qui a repris.*

 Here

Here then we have satisfactory proof of the adhesion of parts of an animal body *totally separated from each other;* and cases are fresh in the memory of most of us, many of which are recorded, of *all but total* separation where the re-union has been complete, nor has the probability of the event given rise to one doubt.

It becomes a question in those cases where a mere thread of skin has been the only remaining medium of connection, whether this is really sufficient to keep up that degree of circulation which has been assumed to be indispensable to reunion—whether this slender connection, which has been supposed capable of preserving the circulation, would be sufficient to keep up the vitality of the separated portion without its apposition and immediate adherence to the part from which it has been removed.

On turning to the 3d volume of the late Dr. Kirkland's Enquiry into the State of Medical Surgery, we find an account of one of these *all but total* separations, which we the more readily transcribe, because it produced an impression on the mind of that excellent and discriminating practitioner of the possibility of uniting parts *totally divided.* So little connection existed between the parts disunited in his case, that they seemed to warrant a belief in the truth of Garangeot's story, or at least of the possibility of the allegations contained in it.

"In the absence of my neighbour, Mr. Cantrell," says Dr. Kirkland, "my nephew Mr. T. Kirkland, and myself, went to Mr. Choice, of Normanton, whose ear was so nearly torn off by a threshing machine as to *hang down merely by skin.* While the parts were cleaning, an idea occurred to me of the possibility of its uniting; and, instead of stripping it off, I desired my nephew to pare off the ragged edges, and lay it again in its place. As it bled when the edges were made even, I had some distant hope that it might succeed. It was therefore surrounded by adhesive plaster and bandage, and left for some days. When Mr. Cantrell thought proper to remove the dressings, he found there was a disposition in some part of it to unite, and though the discharge continued many weeks, it united firmly, and the scar is scarcely observable, the external parts of the ear not having been injured."

Dr. Balfour gives two cases whose authenticity seems unquestionable; since, independent of the authority derived from his personal character, they are ushered into the world with all the weight that judicial deposition and oaths can give them: one occurred in his own family. As they are of too much importance to be trusted to the fleeting pages of a small tract, and may be curious and useful to our numerous readers, we make no apology for transcribing them.

"Case I.—About eleven years ago, Mr. Gordon, surgeon, now, I believe, in India, after having conversed with me for some time one day, in my shop, upon going out, shut the door smartly after him, without perceiving any body near it. Unfortunately, one of my sons, a boy of about four years and a half, diverting himself on on the outside, had one of his hands in the groove of the hinge-side of the door. I was shocked with a wild scream that I heard upon the door being shut; and still more so, when Mr. Gordon came in, carrying the boy in his hands, stretched, from agony, as upon a rack. The points of three of his fingers were completely separated, with the exception of a slight attachment of skin, which barely suspended the parts. The points hung at right angles when the fingers were extended. The point of the index was cut off at the middle of the nail, the fore-finger a little above the nail, and the ring-finger at the root of the nail. The wounded surfaces were necessarily much bruised, but the fingers were, nevertheless, cut so perpendicularly, that, unless I had seen it, I could not have believed a door could have done it. With the assistance of Mr. Gordon, the innocent cause of the accident, I instantly replaced the parts, with but little hopes I confess, owing to the degree of contusion of the wounded surfaces, of reunion taking place. But I was so shocked at the idea of the boy's hand being mutilated for life, that I hesitated not a moment to put the powers of nature to the test. On the sixth day after the accident I removed the bandages, when I found adhesion had taken place, to the unspeakable joy of Mr. Gordon, the boy, and myself. The skin and nails came off all the three fingers, but were afterwards renewed; and the cure was so complete, that a narrow inspection was necessary to discover any difference between the fingers of the one hand and those of the other. There was, indeed, no difference to be perceived, but a slight scar on the left side of the ring-finger, at the root of the nail. This case I certainly would have published at the time it occurred, but on Mr. Gordon's account, who, though not the smallest blame was attributable to him, suffered more anxiety and distress of mind than I did myself, and never liked to hear the subject mentioned. I trust he will now excuse me for mentioning him by name, having no other motive for so doing, than the establishment, beyond the possibility of contradiction, of the truth and accuracy of the above statement. Mr. John Moffat, accountant of Excise, Mr. Alexander Milne, surgeon, now on board the Norge, 74, and my servants, were likewise witnesses of the facts. The boy died of the scarlet-fever, a year and a half after the accident; and but for the following case occurring, which to most, I am sensible, will appear much more interesting and decisive, that of my son would never have been recorded.

Case II.—On the 10th day of June last, two men came into my shop, about eleven o'clock in the forenoon, one of whom, George Pedie, a house-carpenter, had a handkerchief wrapped round his left hand, from which blood was dropping slowly. Upon uncovering the hand, I found one half of the index wanting. I asked him what had become of the amputated part. He told me he had never looked after it, but believed it would be found where the accident happened. I immediately dispatched Thomas Robertson, the man that accompanied the patient, to search for and bring the piece. During his absence I examined the wound, and found that it began near the upper end of the second phalanx, on the thumb side, and terminated about the third phalanx on the opposite side. The amputated piece, as measured by the patient himself, was an inch and a half long on the thumb side, and an inch on the other. The wound was inflicted in the cleanest manner, by one stroke of a hatchet, and terminated in an acute point.

In about five minutes, as nearly as I can guess, Thomas Robertson returned with the piece of finger, which was white and cold; and I remarked to Dr. Reid, who was present, that it looked and felt like a bit of candle. Without the loss of a moment, I poured a stream of cold water on both wounded surfaces, to wash away the blood from the one, and any dirt that might be adhering from the other. I then applied, with as much accuracy as possible, the wounded surfaces to each other, expressing a confident expectation that reunion would take place.

I endeavoured to inspire the patient with the same hopes, by detailing to him the success I had in my son's case, which, for the reasons already mentioned, was to me quite decisive of the question, whether or not parts entirely separated from the system would reunite? All this was heard by the patient with a very apparent distrust. But I could do no more than tell him, that, if reunion did not take place, no harm could ensue from the attempt, and that, if it did, a great deformity would be prevented. I informed him, that unless pain or fetor, or both, should occur, I would not remove the bandages for a week at least; directed him to keep his fore arm slung, and not to think of any kind of work. At last he entered so far into my views as to promise punctual obedience. He called on me next day, when he felt no particular uneasiness, but remarked, that the wound had not altogether given over bleeding. Assuring him there was nothing in that, desired him to call on me every day; but did not see him again till the 4th of July! Concluding, from his absenting himself without assigning any reason, that he was one of those, too frequently to be met with in the lower ranks, who go from one medical man to another, just as their fancy strikes them, or as they happen to be advised by some of their foolish and ignorant neighbours, and whose ingratitude to any practitioner is in exact proportion to the good he does them, I suspected he had fallen into bad hands, and that I never would hear more of him. On the 2nd of July, however, a gentleman called on me, and asked if I recollected a man who had got a finger struck off, about three weeks before, to have come through my hands?

I told him I recollected perfectly well; that I was filled with indignation at the fellow's unreasonable and ungrateful conduct; and that I was just about setting on foot a search after him, not having informed myself either of his name or place where he was employed, at the time he applied to me. The gentleman said he would save me the trouble, for he could give me an account of the man.

The

The accident happened on the 10th of June, and on the 12th the patient, under the influence of the ridicule of his acquaintances, for giving the least credit to my assurances that reunion would take place, applied to another practitioner. This gentleman, I am informed, on being told the object I had in view in replacing the piece of finger, represented the impropriety of any other person intermeddling with it. But, prepossessed with the belief that he carried about a piece of dead matter only tied to the stump of his finger, the man insisted on having the bandages removed, which was done accordingly. Thus were nearly rendered abortive, my attempts at the reunion of the parts, and the profession deprived of a fact, which, as demonstrating the wonderful powers of nature to repair injuries, inferior, in importance, to none in the annals of the Healing Art. But, fortunately, nature had been too busy for even this early interference to defeat her purpose—ADHESION HAD TAKEN PLACE.

In consequence of the information I got from the gentleman who called on me on the 2d of July, I found out the patient on the 4th, when reunion of the parts was complete. The finger, in fact, is the handsomest the man has, and has recovered both heat and sensation. In the progress of the cure, the skin was changed, and, soon after the accident, the nail fell off; but I have not the smallest doubt that it will likewise be renewed.

From the information obtained, not only from the patient himself, but from those present when the accident happened, I am satisfied that upwards of twenty minutes must have elapsed before the parts were replaced. For the patient did not apply to me *immediately* upon receiving the injury. He waited on the spot till a great number of his fellow workmen, separated in different apartments of a large building, came to see and condole with him on the occasion."

Our author, with great show of reason, denies that the circulation is kept up in parts cut out of the forehead, as now practised in India in the making of artificial noses; and he observes, that the *twist* given to the small attachment left at the root of the nose, must almost entirely preclude any thing of the kind; and expresses his conviction, that, had Talicotius at once separated from the system the flaps of skin with which he repaired mutilated parts, his operations would have been equally successful, infinitely less troublesome to himself, and less distressing to his patients.

At the close of this interesting little tract, Dr. B. enters into some speculations respecting the manner in which the reunion is effected; but we are so sick of theories, that we shall not offer one opinion to the notice of our readers. It is proper to add, that the doctor, as a security against the disbelief of his brethren, accompanies his cases with the affidavits of George Pedie, one of the patients, Thomas Robertson, and Dr. Reid, a physician in Edinburgh.

*A View*

*A View of the comparative Advantages and Disadvantages of the Navy and Army Surgeon, and of the Surgeon in Private Practice; together with a proposed Amendment of the Condition of Assistant-Surgeons, at their outset in the Navy.* By WILLIAM CULLEN BROWN, M.D. Ed. Surgeon of his Majesty's Prison-Ship Arve Princen. 8vo.; pp. 80; 1814.

ALTHOUGH a portion of the interest which this production is well calculated to excite, is removed by the recent cessation of hostilities, the continuance of war with America, and the peculiar nature of that war, must still keep up a large naval force, and consequently render the subject interesting to all persons likely to enter the service in a medical capacity. In our opinion it could not have been offered to the public at a more suitable time. In the multifarious business which, during a long and obstinate war, continually occupies the serious attention of the Admiralty, the comforts and convenience of medical officers are not likely to be much attended to; their pay has been increased, and what more ought they to expect? The interval of peace is the season for improvement, and by perusing the interesting pamphlet before us, the Lords of the Admiralty may learn, from an experienced medical officer and an enlightened scholar, the nature of those grievances which, otherwise, it is probable they might never have known or heard of. The author is well qualified for the task he has imposed upon himself, and we observe with pleasure that he has not shrunk from drawing a bold outline of the evils which an assistant-surgeon in the navy endures, nor avoided painting in glowing colours the abuses in the system, which, if not remedied, ought to deter every man of education and principle from exposing himself to. If the acquisition of money is the sole object, indeed, for which young men enter the navy, then the author's statement will produce little effect; but we are of a different opinion, and therefore consider the present publication likely to occasion considerable sensation; it ought to induce young men of liberal education to pause before they subject themselves to a situation degrading and irksome; it ought to induce men in power to obviate the grievances of which it so justly complains.

The author very candidly inquires into the relative advantages of the young surgeon in private practice, in the army, and in the navy, and proves that the latter, in the course of a few years, supposing him to start with equal talents, education, and professional acquirements, becomes decidedly inferior to the two former classes of practitioners. If the facts on which Dr. Brown grounds this assertion be correct, and we have no reason to call them in question, with some happy exceptions, his general principle must be true; it is utterly impossible for young men in the situation of assistant-surgeons in the navy not to suffer from the circumstances in which they are placed.

We shall now present our readers with some extracts, from which they may appreciate the nature of the service which we think Dr. Brown may be instrumental in performing.

"Let

"Let it be taken for granted, then, that the student about to enter into the medical surface of the navy, *at twenty-three years of age* possesses every requisite qualification; that he has been previously well grounded in the classics, and other branches of a liberal education; that he has acquired, under the tuition of the ablest teachers, a reasonably adequate knowledge of the theory and practice of medicine and surgery; that his application to study, his conversation with professional and literary men, as well as with the polite and ingenious of both sexes, in short, that the whole tenor of his education, have formed his character and perfected the gentleman.

"In compliance with the advice of friends, or induced by the naval service, since the late improvement of its medical establishment, now holding forth the fairest prospect of acquiring wealth, or in consequence of the disposition to roam, so natural to youthful and aspiring minds, he determines on going to sea, obtains his warrant, and repairs on-board of his destined ship. How far such change of circumstances is adapted to his preserving unimpaired the knowledge supposed to have been acquired by so much industry, and treasured up at such expense, will appear in the sequel.

"Here all is a scene of novelty to him, calculated to excite his wonder. The manners, the mode of living, and the very language, which prevail on all sides, are matter of surprise to him. Every thing engages his attention and curiosity, and has the effect of banishing the remembrance of the past. This gentleman, so educated, and hitherto accustomed to associate with polite people in middling life, instead of being permitted to mess in the Ward-room, or Gun-room, among officers more suitable to himself in point of years, understanding, and circumstances, and having an established cabin assigned to him, doomed in future to the society of boys and mere striplings, is compelled to submit to the innumerable inconveniences and hardships of the midshipmen's birth, a complete description of which would be neither brief nor easy. The petulance of the younger, and frequently the waggery or rudeness of the older midshipmen, together with the noise of all, effectually combine to prevent him from application; and he can rarely, for an hour together, snatch an opportunity of perusing his book without molestation.

"Denied the privilege of a cabin, or place of refuge from his boisterous messmates, whose clamour and gaiety perpetually stun and distract him, and forced to remain in this degrading situation for two or three years—sometimes much longer,—before he can reasonably expect his promotion, independently of the manifold distresses to which the strange element which he now inhabits at first subjects him;—in such circumstances, what change, at the expiration of three years, is his mind likely to have undergone? Is he become better informed in medicine and surgery? Has he fallen back in his information? Or does he, in this particular, remain stationary? It were wonderful, indeed, if, during so considerable an interval, the original stock of his learning did not suffer material diminution, and if the minutiæ of anatomy, formerly in a great degree familiar to him, as well as his correct knowledge of the symptoms of diseases, did not proportionably escape his memory. It will *now* be found that he can no longer read Horace and Virgil with his former readiness; his mathematics are contracted within the narrow limits of defining a few common axioms; historical facts are recalled by him with difficulty; the practical application of his chemistry is no more familiar to him; the outlines of his botany have faded away: in a word, his memory has become blunted with respect to every branch of human learning. In the mean time, he almost unavoidably has contracted much of the coarseness of manners to which he is hourly exposed; and there is likewise a danger, nor that an imaginary one, that the incessant irksomeness of a situation, from which he cannot extricate himself, and in which he is altogether deprived of any opportunity of resorting to such sources of amusement and refuge from *ennui*, as every well-stored mind must possess, will frequently make him desirous, as it were, of flying from himself, and banishing the habitual uneasiness, or rather wretchedness, of his feelings, by the fatal expedient of having recourse to the bottle. Thus he forgets his urbanity, loses his ambition, becomes indifferent about his profession, and, degenerating into a character little superior in refinement to the seamen around him, takes more interest in the manœuvring of the ship, than in the operations of surgery, or the farther improvement of his mind."

If this be regarded as strong language, let it be remembered that the voice which proclaims the necessity of a reform, must on all occasions be loud and bold: this is not a time for timid hints, and feeble remonstrances—Government have acknowledged the difficulty of supplying the navy with proper medical assistance, they have augmented the pay, and conferred titles of rank on the subordinate officers; but they have omitted to provide for the evils of which Dr. Brown so feelingly complains. We agree with him, that it is a monstrous and unnatural arrangement, that students whose future progress in life, whose fame, wealth, and honour, depend on the full and proper employment of their time, in perfecting what their education has only commenced, should be compelled constantly to associate with a number of wild, thoughtless, giddy, youths, "crowded together (as the author observes) with scarcely any other care than that of satisfying their craving, keeping their watch, and, at night, *turning-in* to their hammocks, enjoying the height of health, and in the hey-day of youth." "They will naturally (he continues) manifest their happiness in boisterous mirth, and in all manner of extravagancies, in practising a thousand tricks on each other, but more especially on the *doctor*, as he is called, whom they look upon as a kind of exotic animal obtruded upon them, of a genius and character altogether foreign to their own, and, consequently, consider in the light of what is familiarly called *fair game*. In particular, there is a general combination against his studying; and the loudest and most successful in obstructing his application, obtains the palm among his confederates."

Amongst such associates, it seems hardly possible for the best disposed disposed and most studious mind to advance in the attainment of knowledge, and it must be extremely difficult for the generality of young men to escape the influence of idle and dissipated habits; so that when the young surgeon has at length obtained the rank for which he aspired, and is appointed full surgeon to a ship, he has lost the habit of studying, and is but ill prepared to fill the situation with honour, or to his own satisfaction.

The sketch which the author draws of the young army surgeon is much more agreeable, and a man must have a very strong bias for the sea, who, after reading it, can have the smallest hesitation in his choice of which service to enter. At the same time we must observe, that even in the land service there are many drawbacks from study, and numerous temptations to dissipation, which can hardly occur in private life. But, if opportunity offer, it is a pleasant, and may be made a most useful, employment of time, for a young man to dispose of two or three years in army practice, before he seriously settles, probably for life, in a narrow sphere of action. He may thus acquire more general knowledge, liberality, and freedom from prejudice.

The author's opinions of private practice are not very favourable. Unquestionably, to minds of a certain cast, nothing can be more irksome or intolerable than the few first years, till the blush of youth is entirely effaced; or, in provincial towns, the jealousies and enmity of *professional friends*, are subdued by steady superiority of conduct or cautious address. The subject is sufficiently hateful, and the author has not attempted to conceal its deformity. If we admired his courage in exposing abuses in the navy, we cannot censure him for lashing the bickering, the servility, and the prejudices, which too frequently disgrace individuals in private practice. We recommend his Treatise to the serious perusal of all young men about entering the medical profession, and parents would do well to read it before they doom their sons to a profession of certain toil and trouble, and doubtful honour and renown. The first lord of the admiralty too, to whom it is dedicated, will do well not to neglect the hints it contains, though we have considerable doubts of the author's being promoted to a higher station. It is not pleasant for an officer to publish to the world that his superiors neglect their duty. We are not now surprised to observe a physician of Dr. Brown's attainments and experience surgeon to a prison-ship, of which, except for his present treatise, we should never have heard.

---

*An Enquiry into the Probability and Rationality of Mr. Hunter's Theory of Life; being the subject of the first two Anatomical Lectures delivered before the Royal College of Surgeons of London.* By JOHN ABERNETHY, F.R.S. &c. Professor of Anatomy and Surgery to the College. 8vo. pp. 95. Longman and Co. 1814.

THE name of John Hunter seems to carry with it a kind of inspiration in the philosophy of surgery. At the sound of it, all exclaim *Jovis omnia plena*. Yet it is difficult to say whether he is more praised or abused. Among his countrymen, he has rarely fair play allowed him. If we attend to the Bells, men of no mean consideration, we shall find nothing but obscurity, affectation, and error, in whatever is quoted from the subject of this panegeric. Not to mention Mr. Jesse Foot, it would be easy to produce many other writers who have been anxious to find errors in so conspicuous a character, and even his biographer Sir Everard Home has not been backward in relating many cases in which it was necessary for him (Sir Everard) to repair his master's errors.*

When we recollect all these circumstances, and compare them with the increasing celebrity of Mr. Hunter, we cannot but view in a still more exalted light the extraordinary merits of the man whose fame only increases as the science of medicine advances; yet, on a fair estimate of the question, may we not be allowed to ask, where shall we find an intelligible statement of his doctrines? Not in his writings, for, after the utmost diligence, (and the nature of our occupation imposes much industry upon us,) we confess that we have never risen from a perusal of his most laborious works with either satisfaction or improvement. The heaviness of style, the tedious detail of experiments, and the obscurity of terms, require attention to such a variety of points at the same time, as always fatigues us before we can gain sufficient information. We are not ignorant how much we owe to Dr. Adams for rendering Mr. Hunter's only practical work intelligible to us, but still, in the introductory commentaries to the Treatise on the Venereal Disease, the grand theories are no further explained than the subject required; and we have never met with any thing in the various encomiums bestowed by that able writer on a master whom he seems to idolize, which could seem even to hint at Mr. HUNTER'S THEORY OF LIFE.

On this account we seized with eagerness the opportunity of examining the work before us: for though we were aware there is on some occasions a want of perspicuity in Mr. Abernethy's writings, yet we could not doubt that we should meet with a satisfactory explanation of the only subject proposed in the title-page.

Mr. Abernethy's character stands too high to require any apology from us. Besides which, from the nature of our publication, if we have misconceived or misrepresented him, it is always in his power, or in the power of his friends, to rectify our errors; and nothing, we are sure, will give our readers more pleasure, than to find such a subject elucidated even at the expense of exposing our dullness. We shall therefore go through the pages as they occur, marking only the impression they leave on our minds, and waiting to find that impression confirmed or removed.

The exordium contains a compliment to Sir William Blizard, who, it appears, was Mr. Abernethy's earliest instructor in these sciences, and whose moral precepts were not less valuable than his anatomical lessons. After this we have some remarks on the importance of truth, and the value of the healing art: in these, much

---

* See Treatise on Stricture *passim*.

novelty novelty cannot be expected. Sir Everard Home next receives a complimentary apostrophe, as one who pursues the path of science pointed out by Mr. Hunter. Mr. A. expresses his wish to do the same, and also to engage the attention of his hearers on the *probability and rationality of Mr. Hunter's Theory of Life.* A definition of theory and hypothesis follows, and we are told that the facts collected by Mr. Hunter seem sufficient to establish his *opinions* respecting life, on which account they are considered to deserve the name of *theory.* A number of remarks follow on the subject of theory, as distinct from hypothesis and opinion; and, after the second introduction of Sir Isaac Newton's name, we are told, "Our theories, hypotheses, or opinions, for to me all these words seem to refer to one act of the mind," &c.—Hypothesis and opinion we are ready to admit to be nearly the same, but there is a difference, which the etymology readily points out: supposition is the literal Latin translation of the Greek word *hypothesis;* but the etymology, as well as the received meaning, of theory, differs from hypothesis and opinion. The terms, like most others expressive of intellectual objects, are borrowed from such as are applied to the external senses, that is, we express by the word *theory* what is so satisfactory to the understanding, that we *see* it with the mind's eye; and such, we conceive, must be Mr. Abernethy's meaning, when he afterwards says, that an hypothesis may be converted into a theory, that is, we conceive, that, when proved, what was at first only *supposed* is *seen* and universally admitted.

If, in tracing the next division of the harangue, we should not perfectly understand Mr. Abernethy, we shall at least be careful not to misrepresent him. We are first told, "that it is by means of the organization of the body the mind acquires all its information;" and in the next paragraph, "When we find that every organ and every portion of it is composed of a few *and simple fibres;* that by these it *is originally formed,* kept in constant repair, and endowed with *animation,* sensation, and motion." Here, we must first remark, we are "lost in astonishment," as the author expects us to be; but a part of that astonishment is what the orator can mean, and next, in what part of Mr. Hunter's writings such opinions can be found. We should indeed have conceived that the illustrious dead had been entirely forgotten, and that Mr. A. was now proceeding with his own opinions, were it not that the paragraph, which finishes with a pause, closes with the following sentence: "I shall begin with the fibres, the only visible means by which motion and sensation are *produced;* for this will lead directly to the *consideration of Mr. Hunter's theory of life.*

The oration is resumed with the following paragraph, which we transcribe, because it contains more about Mr. Hunter than any *other passage.*

"In surveying the great chain of living beings, we find life connected with a vast variety of organization, yet exercising the same functions in each; a circumstance from which we may, I think, naturally conclude, that life does not depend on organization. Mr. Hunter,

Hunter, who so patiently and accurately examined the different links of this great chain, which seems to connect even man with the common matter of the universe, was of this opinion. In speaking of the properties of life, he says, it is something that prevents the chemical decomposition, to which dead animal and vegetable matter is so prone; that regulates the temperature of the bodies it inhabits, and is the cause of the actions we observe in them. All these circumstances, though deduced from an extensive contemplation of the subject, may, however, be legitimately drawn from observations made on the egg. A living egg does not putrefy under circumstances that would rapidly cause that change in a dead one. The former resists a degree of cold that would freeze the latter; and, when subjected to the genial warmth of incubation, the matter of it begins to move or to be moved so as to build up the curious structure of the young animal."

After the former remarks, we leave our readers to determine the meaning of the commencement of this paragraph, and shall only observe, that we are ready to admit that life is not the effect of organization, yet we cannot say that it is entirely independent of it, when we know by experience that the disorganization of certain parts is sufficient to destroy life in the whole. The subsequent remarks only relate to Mr. Hunter's definition of life, but say nothing concerning his theory of life. However we are pleased with this part of the orations, as it proves the means of introducing a pithy little sentence, which, though not entirely new, we feel an irresistible inclination to transcribe.

"Many persons (says Mr. A.) have genius without industry; others industry without genius; and *many who have both are still deficient in judgment.*" Some remarks on genius and the other properties of the mind follow; after which we are again informed that the orator now proceeds "to consider the structure and functions of those fibres which constitute the muscles, in order to introduce the discussion of *the probability and rationality of Mr. Hunter's theory,* as a cause of irritability." A minute and very judicious description of muscular fibres follows, with some remarks on muscular motion. The constant action of the sphincters, and the contraction of muscles, from powerful stimuli after apparent death, which, after a short cessation, may be again excited, induce our author to conclude that "the foregoing facts appear to show the impropriety of the phrase exhausted irritability, *which is in common use.*" We should be glad to know with whom, excepting the few remaining followers of Brown and Darwin.

The subject of muscular contraction is continued without any novelty till we are brought to "review the conjectures that have been formed as to the cause of these curious, sudden, and powerful contractions," by which we suppose are meant muscular contraction in general. Without attending to exploded hypotheses, only the more modern ones are introduced.

The first of these is, that "the contraction is the effect of chemical change occurring in the part." This we conceive connected with Mr. Home's opinion that secretion may be the effect of galvanism. However that may be, we shall not entertain so little respect for our readers as to conceive it necessary to combat such phantoms.

Secondly, we are told that contraction of irritability is supposed to be a *property* of the muscular fibre: of this, as it occurs again, we shall say more hereafter.

Thirdly, we are referred to Mr. Hunter's opinion, "that irritability is the effect of some subtile, mobile, invisible substance, superadded to the evident structure of muscles, or other forms of vegetable and animal matter, as magnetism is to iron, and as electricity is to various substances with which it may be connected. Mr. H. doubtless thought, and I believe most persons do think, that, in magnetic and electric motions, a subtile invisible substance, of a very quickly and powerfully mobile nature, puts in motion other bodies which are evident to the senses, and are of a nature more gross and inert. To be as convinced as I am of the probability of Mr. Hunter's theory as a cause of irritability, it is, I am aware, necessary to be as convinced as I am that electricity is what I have now supposed it to be, and that it pervades all nature. To obtain this conviction, it is necessary that the facts connected with this subject should be attentively considered; but for such an examination I have no time; neither would it be considered as suitable to the general design of these lectures."

Some remarks follow on the properties of matter, and afterwards of electricity. The phenomena of electricity and life, we are told, correspond. "Electricity may be attached to, or inhere in, a wire." And is life attached to, or does it inhere in, a wire? "So life (continues Mr. A.) inheres in vegetables and animals." What can this analogy mean: is life inhesion then?

"The motions of electricity are characterised by their celerity and force; so are the motions of irritability. The motions of electricity are vibratory; so likewise are those of irritability. When by long continued exertion the power of muscles is fatigued, or when it is feeble, their vibratory or tremulous motions are manifest to common observation, but the same kind of motion may be perceived at all times by attention, as has been shewn by Dr. Woolaston in the Croonian Lecture for the year 1810. It is then, I think, manifest that Mr. Hunter's conjectures are the most probable of any that have been offered as to the cause of irritability."

We confess ourselves greatly at a loss to understand this; and unfortunately the time allotted does not permit Mr. Abernethy to consider the other vital functions, a consideration which he relinquishes with reluctance, because he has been speaking only on that point in which it seems most difficult to persuade the incredulous of *the probability and rationality of Mr. Hunter's Theory.* If his time was short in the theatre, we cannot avoid expressing a wish he had allowed himself more before he went to press.

After another pause, a very eloquent paragraph follows, in which we are informed that "Mr. Hunter points out to us how matter starting starting from the general mass springs into life in vegetation." We confess we should have been much less surprised to have met with such a passage in Darwin or any other poet; though these gentlemen for the most part invoke the assistance of some Muse before they engage in so arduous an undertaking. We are next informed that the torpedo and gymnotus have *organs* forming an electric battery; but, as in the next page it is observed that Mr. Hunter always considered life as something independent of *organisation,* this does not yet lead us nearer to his theory. Nor can we find any advantage derived from the experiments of Sir Humphrey Davy; for, though that acute philosopher pointed out to us that electricity is sufficient to produce those chemical changes we perceive in inert matter, without the intervention of human aid, yet this does not in the least lead to Mr. Hume's suggestion that secretion should be the effect of a similar cause. We therefore earnestly hope that in a future edition Mr. Abernethy will revise the following short paragraph.

"When, therefore, we perceive in the universe at large a cause of rapid and powerful motions of masses of inert matter, may we not naturally conclude the inert molecules of vegetable and animal matter may be made to move in a similar manner by a *similar cause.*"

The reader will with us perhaps suppose that by this is meant that the change from dead animal and vegetable matter to life is induced by electricity. We are, however, assured that "it is not meant to be affirmed that electricity is life, only that *Mr. Hunter's theory is verifiable* that there is a subtle substance of a quickly powerful and mobile nature pervading every thing appearing to be the life of the world, and therefore that it is probable that a similar substance pervades organized bodies, and produces similar effects." Sir H. Davy's experiments seem, we are told, to realize the speculations of philosophers concerning the *anima mundi.* Pythagoras and Virgil are made to speak a similar language, but Mr. Abernethy chuses rather to adopt the words of Lucretius:

> Inde hominum pecudumque genus, vitæque volucrum

> Et quæ marmoreo fert monstra sub æquore pontus.

Thus poetically ends the first Lecture. In the second, Mr. A. proceeds to speak of the structure and functions of the nervous fibres. Their divisions, ramifications, and plexuses, are hinted at in a cursory manner, only with a view to the consideration of Mr. Hunter's Theory of Life, which now seems seriously entered upon: we shall therefore quote the following paragraphs:

"First, then, it is generally believed that all sensation is in the brain, and that all volition proceeds from that organ. This proposition requiring to be impressed so as to produce conviction, for it is the foundation on which all our future reasoning is founded, I shall state the principal causes of this opinion. First, If the continuity of a nerve be intercepted at any point between that extremity which receives impressions from the objects of sense, and which therefore may be called the impressible or tangible extremity, and that that which communicates with the brain, and is usually called its sensorial extremity, both feeling and volition by means of that nerve are suspended.

"2dly. If a certain degree of pressure be made upon the brain, both feeling and voluntary motion cease whilst it continues, and return when it is removed.

"3dly. As we have evidence that the perceptions and intellect of animals increase in proportion as the brain becomes larger and more complex, so we have reason to conclude that these faculties are connected with that part of the nervous system.

"4thly. The conviction which we generally though not constantly experience, that feeling exists in the part which receives impressions, is shewn to be deceptive by the following facts. If a nerve be irritated midway between the brain and its extremities, severe pain is supposed to be felt in those extremities; and if it supplies muscles, those muscles become convulsed. Thus, when a disease forms about the hip joint, or in the loins, many persons have applied poultices to their knees, from a conviction that as the pain was felt in the knee, it was the seat of the disorder. In like manner, persons who have had their limbs amputated, can scarcely believe that they are removed, because of the pain and other sensations they still seem to feel in them. In either of these cases, motions being excited in the middle of nerves, and transmitted to the brain, are attributed to a disordered state of those parts, from which such motions have heretofore originated."

In these paragraphs we meet with nothing particularly objectionable, but they are neither discoveries of Mr. Hunter, nor has he ever claimed them as such, nor, as far as we know, built any theory upon them. The theory of a nervous fluid might be Baron Haller's, and perhaps the Latin quotation from that author may express as much; but we confess that either the Latinity or philosophy is so obscure as to baffle all our attempts at understanding him. On this account, we could wish Mr. A. had given us an English translation of the passage. Most of all, however, we wish, as Mr. Hunter always wrote in our mother tongue, that Mr. A. had been as careful to produce his words as he has the words of many others. We are told "Mr. Hunter's opinion of a subtle and mobile substance inhering in the nervous chords, is not essentially different from that of Haller." Let us then know precisely what Haller's is, and let us be informed of the passage in which Mr. Hunter speaks of this subtle and mobile fluid. We do not recollect to have seen it in his works. If contained in a MS. copy of his lectures, this would have been sufficient authority with us, had the passage been stated at length, with the name of the copyist.

We are now informed (page 79) that the first lecture contained the arguments to show that *Mr. Hunter's theory of life is verifiable.* Already we have lamented that we have not learned what the theory is, consequently are little able to judge how far it is verifiable. Now we are told that his theory of irritability does not materially differ from the theory of the best pathologists. But what can be meant by "irritability as a *vis insita* of the muscles independent of the *vis nervea.*" Can we entirely detach a muscle from every filament of nerves; or, if we could, can we call that a vis insita which only remains as long as life continues? It is said that this opinion of vis insita has received additional corroboration from Mr. Brodie's experiments: we should be glad to know from which? The succeeding observations are neither new nor at all objectionable; after which follows a passage, the inferences from which seem entirely at variance from all we have ever heard of Mr. Hunter's opinions.

"Assuredly, motion does not necessarily imply sensation: it takes place where no one ever yet imagined there could be sensation. If I put on the table a bason containing a saturated solution of salt, and throw into it a single crystal, the act of crystallization would begin from the point touched, and rapidly and regularly pervade the liquor till it assumed a solid form. Yet I know I should incur your ridicule, if I suggested the idea that the stimulus of the salt had primarily excited the action, or that its extension was the effect of continuous sympathy. If also I threw a spark amongst gunpowder, what would you think were I to represent the explosion as a struggle resentful of injury, or the noise as the clamorous expression of pain?"

Only one answer can be made to all this. It would be ridiculous to impute the crystallization of salts to the same cause as produces the spreading of inflammation in an animal body, and not less so to impute the explosion of gunpowder to a sense of pain in its ingredients. But are not these events uniform and constant? Do not the application of the same external causes invariably produce the same effect in both? Is such the case with the causes of inflammation in an animal body? Do they not vary in different animals of the same species, and even in the same animal at different times? Does not inflammation cease, and do not the parts restore themselves after the mischief thus produced; and are they not again liable to similar effects? Is such the case with gunpowder, or in the crystallization of salts, till we renew an artificial process; and is there any thing in animal life which we can uniformly and artificially restore.

At length Mr. A. regrets that the compass of a lecture is too short to establish *the rationality of Mr. Hunter's Theory of Life.* He however proceeds to remark, that "if it be not electricity, at least we have reason to believe it of a nature similar, and has the power of regulating electrical operations." This analogy the author conceives proved by the power which electricity possesses of altering the temperature of body from the freezing of water to the fusion of platina. Lastly, he observes, that "the varying and strong retention of life by seeds, and some kinds of vegetables and animals, are facts which seem more satisfactorily solved by *Mr. Hunter's Theory of Life*, than by any other."

Such is the general outline of two Lectures delivered before a Royal College of Surgeons in the first metropolis in the world. Such is given by a teacher of anatomy at the first Royal Hospital in the the same metropolis, as a description or rather defence of the physiological opinions of a name not less the glory of British medicine, than Newton is of philosophy in general. No one is more ready to render due honour to so illustrious a name than ourselves: it must therefore be painful to us if we cannot comprehend what we wish to praise.

